# Nitric Oxide Mediates 5-Aminolevulinic Acid-Induced Antioxidant Defense in Leaves of *Elymus nutans* Griseb. Exposed to Chilling Stress

**DOI:** 10.1371/journal.pone.0130367

**Published:** 2015-07-07

**Authors:** Juanjuan Fu, Xitong Chu, Yongfang Sun, Yanjun Miao, Yuefei Xu, Tianming Hu

**Affiliations:** 1 Department of grassland science, College of Animal Science and Technology, Northwest A&F University, Yangling, Shaanxi, China; 2 College of Plant Science, Tibet Agriculture and Animal Husbandry College, Linzhi, Tibet 860000, China; Zhejiang University, CHINA

## Abstract

Nitric oxide (NO) and 5-aminolevulinic acid (ALA) are both extremely important signalling molecules employed by plants to control many aspects of physiology. In the present study, the role of NO in ALA-induced antioxidant defense in leaves of two sources of *Elymus nutans* Griseb. (Damxung, DX and Zhengdao, ZD) was investigated. Chilling stress enhanced electrolyte leakage, accumulation of malondialdehyde (MDA), hydrogen peroxide (H_2_O_2_) and superoxide radical in two *E*. *nutans*, which were substantially alleviated by exogenous ALA and NO application. Pretreatment with NO scavenger PTIO or NOS inhibitor L-NNA alone and in combination with ALA induced enhancements in electrolyte leakage and the accumulation of MDA, H_2_O_2_ and superoxide radical in leaves of DX and ZD exposed to chilling stress, indicating that the inhibition of NO biosynthesis reduced the chilling resistance of *E*. *nutans* and the ALA-enhanced chilling resistance. Further analyses showed that ALA and NO enhanced antioxidant defense and activated plasma membrane (PM) H^+^-ATPase and decreased the accumulation of ROS induced by chilling stress. A pronounced increase in nitric oxide synthase (NOS) activity and NO release by exogenous ALA treatment was found in chilling-resistant DX plants exposed to chilling stress, while only a little increase was observed in chilling-sensitive ZD. Furthermore, inhibition of NO accumulation by PTIO or L-NNA blocked the protective effect of exogenous ALA, while both exogenous NO treatment and inhibition of endogenous NO accumulation did not induce ALA production. These results suggested that NO might be a downstream signal mediating ALA-induced chilling resistance in *E*. *nutans*.

## Introduction

Low temperature is one of severe environmental stress that limits plant growth, development, survival and crop productivity [1]. Exposure to chilling stress disrupts the metabolic balance of cells, inducing the enhanced production of reactive oxygen species (ROS), such as hydrogen peroxide (H_2_O_2_), superoxide radical (O_2_^•−^), hydroxyl radical (HO^•^) and singlet oxygen ($o_{2}^{1}$), leading to oxidative damage [2]. Oxidative stress may be a significant factor involved in chilling damage [3]. Plants have evolved efficient antioxidant defense systems that protect them from the damaging effects of chilling stress. These antioxidants include the enzymes, ascorbate peroxidase (APX), superoxide dismutase (SOD), glutathione reductase (GR) and monodehydroascorbate reductase (MDHAR), the non-enzymatic antioxidants such as reduced ascorbate (AsA), glutathione (GSH) and flavonoids, proline, carotenoids and tocopherols [4, 5]. Moreover, the generation of ROS is increased with the enhancement in antioxidant enzyme activities and antioxidant concentrations, indicating that the antioxidant defense system may play a pivotal role in the improvement of resistance in plants under stress conditions such as chilling stress [6].

Plant growth regulators (PGRs) play an important role in the physiology of plants which include the uptake of nutrients, stomatal movement, photosynthesis and activities of different enzymes under the different stress conditions [7, 8]. One of these PGRs is 5-aminolevulinic acid (ALA), which is also believed to be an essential biosynthetic precursor for the biosynthesis of tetrapyrrols such as heme and chlorophyll [9]. It has been shown that high concentrations of ALA are used as herbicides or pesticides [10]. However, ALA at low concentration enhances the chlorophyll biosynthesis and photosynthesis of crops, thus regulating the growth and development of plants and increasing the yield of crops [11]. Accumulating evidence indicates that exogenous application of ALA regulates antioxidant enzyme activities [12] and thereby increases the resistance of plants to stresses of low temperature [13, 14], heat [15], low light [16], herbicide [17], salinity [18] and heavy metal stress [19], indicating that ALA can be used to mitigate environmental stresses. Liu et al. [20] also found that ALA at 0.1–10 mg/l can improve the GSH level, a total glutathione contents and GSH/GSSG ratio in oilseed rape seedlings under water-deficit stress. Although previous study showed that treatment with ALA resulted in the enhancement in chilling resistance of soybean plants by up-regulating of heme-based antioxidant enzyme [13], the protective mechanisms of ALA in plants under chilling conditions are still limited.

Nitric oxide (NO), a reactive nitrogen species (RNS), is a gaseous signal molecule involved in plant various physiological processes, including plant growth, development and abiotic stresses such as UV-B, salinity, drought, light, extremely temperature and heavy metal stress [21–26]. NO may protect plants against oxidative stress by acting as an antioxidant directly scavenging the ROS generated under stress conditions or operating as a signal molecule in the cascade of events leading to gene expression [27]. Accumulating evidence indicates that NO serves as a signal in developmental, hormonal, and environmental responses in plants [28, 29]. However, whether NO is involved in ALA-induced antioxidant defense remains to be determined.

In the present study, pretreatment with the NO scavenger PTIO and NOS inhibitor L-NNA to investigate whether NO is involved in ALA-induced chilling resistance and ALA-mediated ROS levels, antioxidant defense and the activation of PM H^+^-ATPase in the leaves of two *E*. *nutans* exposed to chilling stress. Our results showed that NOS-dependent NO generation acts downstream of ALA mediating chilling stress induced oxidative damage by the up-regulation of antioxidant enzyme activities and the activation of PM H^+^-ATPase.

## Material and Methods

### Plant materials and treatments

*Elymus nutans* seeds were obtained from two sources: seeds of Damxung (DX) were collected in September 2012, from wild plants growing in Damxung County (30°28.535′N, 91°06.246′E, altitude 4678 m), belonged to Agriculture and Animal Husbandry Bureau in Tibet, located in the middle of Tibet, China. This study was supported by National Natural Science Foundation of China (No. 31402129), which was a cooperation research program by Agriculture and Animal Husbandry Bureau in Tibet and Northwest A&F University. We obtained permission for our field study from Agriculture and Animal Husbandry Bureau in Tibet. *E*. *nutans* occurs naturally and abundantly at altitudes between 3,000 and 5,000 m in the Qinghai-Tibetan Plateau, the field studies did not involve endangered or protected species. And Zhengdao (ZD) seeds were obtained in September 2012, from Beijing Rytway Ecotechnology Co., Ltd., located in Changping District (40°06.595′N, 116°24.383′E, altitude 550 m), Beijing, China.

Seeds were cleaned and stored at 4°C in paper bags until the start of the experiments. In the previous study, two sources of *E*. *nutans*, DX and ZD, were found that ZD was more susceptible to cold stress than DX [30]. Seeds were surface sterilized in 0.1% (w/v) sodium hypochlorite, rinsed several times in distilled water, and germinated on moistened filter paper for 7 d. Morphologically uniform seedlings were selected and plugged into plate holes on plastic pots (five plants per pot). All seedlings were cultured in silica sand by irrigating 10 ml Hoagland nutrient solution once every 3 d. Plants were germinated and grown in a growth chamber at a day/night temperature 5/5°C, a relative humidity of 70%, a day/night regime of 12/12 h and a photosynthetic photon flux density (PPFD) of 100 μmol m^-2^ s^-1^. Light was provided by a fluorescent lamp (Philips Electronics N. V. Holland, Nanjing, China). After 4 weeks growth, plants were treated by foliar spray with 15 ml different concentrations of SNP, ALA, PTIO, L-NNA after automatically turning off the light in the chamber according to the program-set for photoperiod (12/12 h), as described earlier. Control seedlings were treated with the same volume of distilled water. After 12 h of pretreatments, the plants were subjected to chilling stress at 5°C for 5 d under the same light regime as indicated above [31]. Plants not exposed to chilling stress and grown in the growth chamber at 25°C were used as the control (and identical the rest of growth conditions). After 5 d of chilling stress, the seedlings (*n* = 3 of per treatment) were harvested and frozen in liquid nitrogen, then stored at -80°C for further bio-chemical and physiological measurements.

### Assay of morphological parameters

At the end of the experiments, 5 healthy plants were randomly chosen from each group. The shoots of the seedlings were cut at the growth medium line. The shoots were dried at 80°C for 72 h and their dry weights were determined.

### Determination of electrolyte leakage and lipid peroxidation

Electrolyte leakage was determined by the modified method according to Song et al. [32]. The fresh leaves (0.5 g) were washed in deionized water and placed in Petri dishes with 5 ml deionized water at 25°C for 2 h. After the incubation, the conductivity was measured (EC_1_). Then, the samples were boiled for 20 min and conductivity was read again (EC_2_). The electrolyte leakage was calculated as EC_1_/EC_2_ and expressed as percent.

Membrane lipid peroxidation was measured as the concentration of malondialdehyde (MDA) produced using 10% (w/v) trichloroacetic acid (TCA), according to Dhindsa et al. [33]. The absorbance of the supernatant was measured at 450, 532, and 600 nm.

### Measurement of hydrogen peroxide and superoxide radical

Hydrogen peroxide concentration was measured according to Liu et al. [34]. Leaves were homogenized in cold acetone and centrifuged at 3,000 × *g* at 4°C for 10 min. The supernatant was mixed with titanium reagent (prepared in concentrated hydrochloric acid containing 20% (v/v) titanium tetrachloride), and then ammonium hydroxide was added to precipitate the titanium-peroxide complex. The reaction mixture was centrifuged at 16,000 × *g* at 4°C for 10 min, and the pellet was washed with cold acetone. The pellet was dissolved in 1 M H_2_SO_4_. The absorbance of the solution was measured at 410 nm. H_2_O_2_ concentrations were calculated using a standard curve prepared with known concentrations of H_2_O_2_.

Superoxide radical (O_2_^•-^) production rate was determined by the modified method according to Xu et al. [35]. One milliliter of 1 mM hydroxylammonium chloride was added to 0.5 ml of the supernatant and incubated for 1 h at 25°C. The addition of 1 ml 4-aminobenzenesulfonic acid (17 mM) and 1 ml anaphthylamine (7 mM) for 20 min at 25°C altered the color, and the specific absorption at 530 nm was determined. Sodium nitrite was used as a standard solution to calculate the O_2_^•-^ levels.

### Quantification of non-enzymatic antioxidant concentrations and antioxidant enzyme activities

Reduced glutathione (GSH) and oxidized glutathione (GSSG) concentrations were determined according to Law et al. [36] with some modifications. Leaves (0.3 g) were homogenized with 5 ml of 10% (w/v) TCA and homogenate was centrifuged at 15,000 × g for 15 min. To assay total glutathione, 150 μl supernatant was added to 100 μl of 6 mM 5,5′-dithio-2-nitrobenzoic acid (DTNB), 50 μl of glutathione reductase (10 units ml^-1^), and 700 μl 0.3 mM nicotinamide adenine dinucleotide phosphate (NADPH). The total glutathione content was calculated from the standard curve. All the reagents were prepared in 125 mM NaH_2_PO_4_ buffer, containing 6.3 mM ethylene diamine tetraacetic acid (EDTA), at pH 7.5. To measure GSSG, 120 μl of supernatant was added to 10 μl of 2-vinylpyridine followed by 20 μl of 50% (v/v) triethanolamine. The solution was vortex-mixed for 30 s and incubated at 25°C for 25 min. The mixture was assayed as mentioned above. Calibration curve was developed by using GSSG samples treated exactly as above and GSH was determined by subtracting GSSG from the total glutathione content.

After 0.2 g of leaves was suspended in 3 ml of 6% TCA and was centrifuged at 4°C and 15,000 × g for 20 min, the contents of ascorbic acid (AsA) and total ascorbate were assayed at 525 nm [37]. The difference between the levels of total ascorbate and AsA was used for estimating the content of oxidized ascorbate.

The leaves (0.5 g) were homogenized with a mortar and pestle at 4°C in 5 ml 50 mM phosphate buffer (pH 7.8) containing 1 mM EDTA and 2% PVP. Homogenate was centrifuged at 12,000 × g for 20 min at 4°C and the supernatant was used for enzyme activity assays. Protein content in the supernatant was determined according to the method of Bradford [38] with bovine serum albumin (BSA) as standard.

The assay for ascorbate peroxidase (APX) activity was measured in a reaction mixture of 3 ml containing 100 mM phosphate (pH 7), 0.1 mM EDTA-Na_2_, 0.3 mM ascorbic acid, 0.06 mM H_2_O_2_ and 100 μl enzyme extract. Change in absorption was observed at 290 nm 30 s after addition of H_2_O_2_ [39]. One unit of APX forms 1 μM of ascorbate oxidized per minute under assay conditions. The activity of catalase (CAT) was measured by following the consumption of H_2_O_2_ at 240 nm according to Cakmak and Marschner [40]. The decrease in the absorption was followed for 3 min and a breakdown of 1.0 μM H_2_O_2_ ml^-1^ min^-1^ was defined as 1 Unit of CAT activity. Glutathione reductase (GR) activity was measured by following the decrease in absorbance at 340 nm due to NADPH oxidation. The reaction mixture contained tissue extract, 1 mM EDTA, 0.5 mM GSSG, 0.15 mM NADPH and 50 mM Tris–HCl buffer (pH 7.5) and 3 mM MgCl_2_ [41]. The reaction was started by using NADPH. Activity of superoxide dismutase (SOD) was determined according to Beauchamp and Fridovich [42] by following the photo-reduction of nitroblue tetrazolium (NBT) at 560 nm. One Unit of SOD activity was defined as the amount of enzyme required to cause a 50% inhibition of NBT reduction.

### Assay of plasma membrane (PM) H^+^-ATPase activity

Plasma membrane vesicles were isolated from fresh leaves by phase partitioning according to the procedure by Palmgren et al. [43]. PM H^+^-ATPase activity was measured according to the procedure of Ahn et al. [44].

### Determination of NO production and NOS activity

NO content determination was performed according to Murphy and Noack [45] with some modifications. The fresh leaves (0.5 g) were incubated with 100 units of catalase and 100 units of SOD for 5 min to remove endogenous ROS before addition of 5 ml oxyhaemoglobin (5 mM). After 2 min incubation, NO concentrations were estimated by following the conversion of oxyhaemoglobin to methaemoglobin spectrophotometrically at 577 and 591 nm. NOS activity was determined according to the method of Murphy and Noack [45].

### Quantification of ALA concentration and 5-Aminolevulinic acid synthetase (ALAS)

Leaves (0.1 g) were homogenized in 2 ml of 1 M sodium acetate buffer (pH 4.6) and centrifuged at 12,000 × g for 10 min. The assay mixture consisted of 0.1 ml of supernatant, 0.4 ml of distilled water, and 25 μl of acetylacetone. The assay medium was mixed and heated in a boiling water bath for 10 min. The extract was then cooled at room temperature, and an equal volume of modified Ehrlich’s reagent was added and vortexed for 2 min. After 10 min of incubation, absorbance of the extract was measured at 555 nm and ALA content was determined from the standard curve of ALA [46]. The activity of ALAS was assayed according to the methods of Hampp et al. [47].

### Statistical analysis

Each experiment was repeated at least three times. All values were expressed as means ± SD. Statistical analyses were performed by analysis of variance (ANOVA) using SPSS-17 statistical software (SPSS Inc., Chicago, IL, USA). Means were separated using Duncan’s least significance difference test at *P <* 0.05.

## Results

### ALA alleviates chilling stress–induced growth inhibition

**Fig 1:**
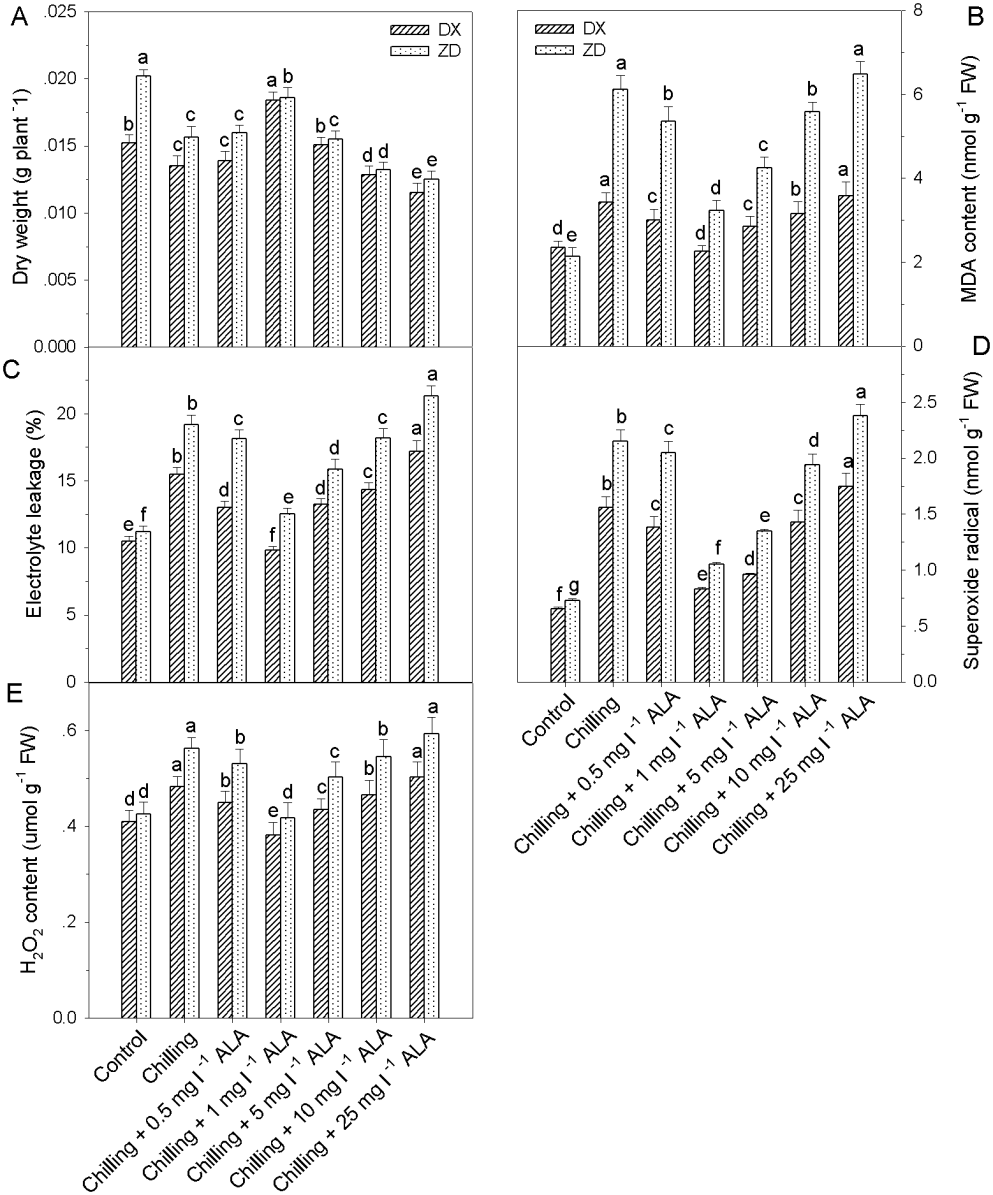
Effects of different ALA concentrations (0, 0.5, 1, 5, 10 and 25 mg l^-1^) on plant growth and cellular membrane damage of *E*. *nutans* (DX, Damxung and ZD, Zhengdao) seedlings under chilling stress (5°C). Plants treated with distilled water under the normal conditions (25°C) served as controls. Each value represents the mean ± SD. Each experiment was repeated at least three times. Bars with different letters are significantly different at the 5% level.

Chilling stress resulted in significant growth suppression in two *E*. *nutans* seedlings, with dry weight decreasing by 22% and 11% of the control in ZD and DX, respectively (Fig 1). A biphasic response in the changes of dry weight was observed in both *E*. *nutans* plants exposed to 0.5–25 mg l^-1^ ALA treatment and cold stress, with the first increase occurring after 0.5 mg l^-1^ ALA treatment and a maximum being reached after 1 mg l^-1^ ALA treatment, but higher concentration of ALA (≥5 mg l^-1^) reduced the dry weight.

### ALA mitigates chilling stress-induced oxidative damage

ALA concentrations from 0.5 mg l^-1^ to 5 mg l^-1^ alleviated electrolyte leakage, MDA, H_2_O_2_ and superoxide radical, with 1 mg l^-1^ ALA prior to chilling stress being most effective (Fig 1). As a result, 1 mg l^-1^ ALA was applied in subsequent experiments.

### NO alleviates chilling stress–induced growth inhibition

**Fig 2:**
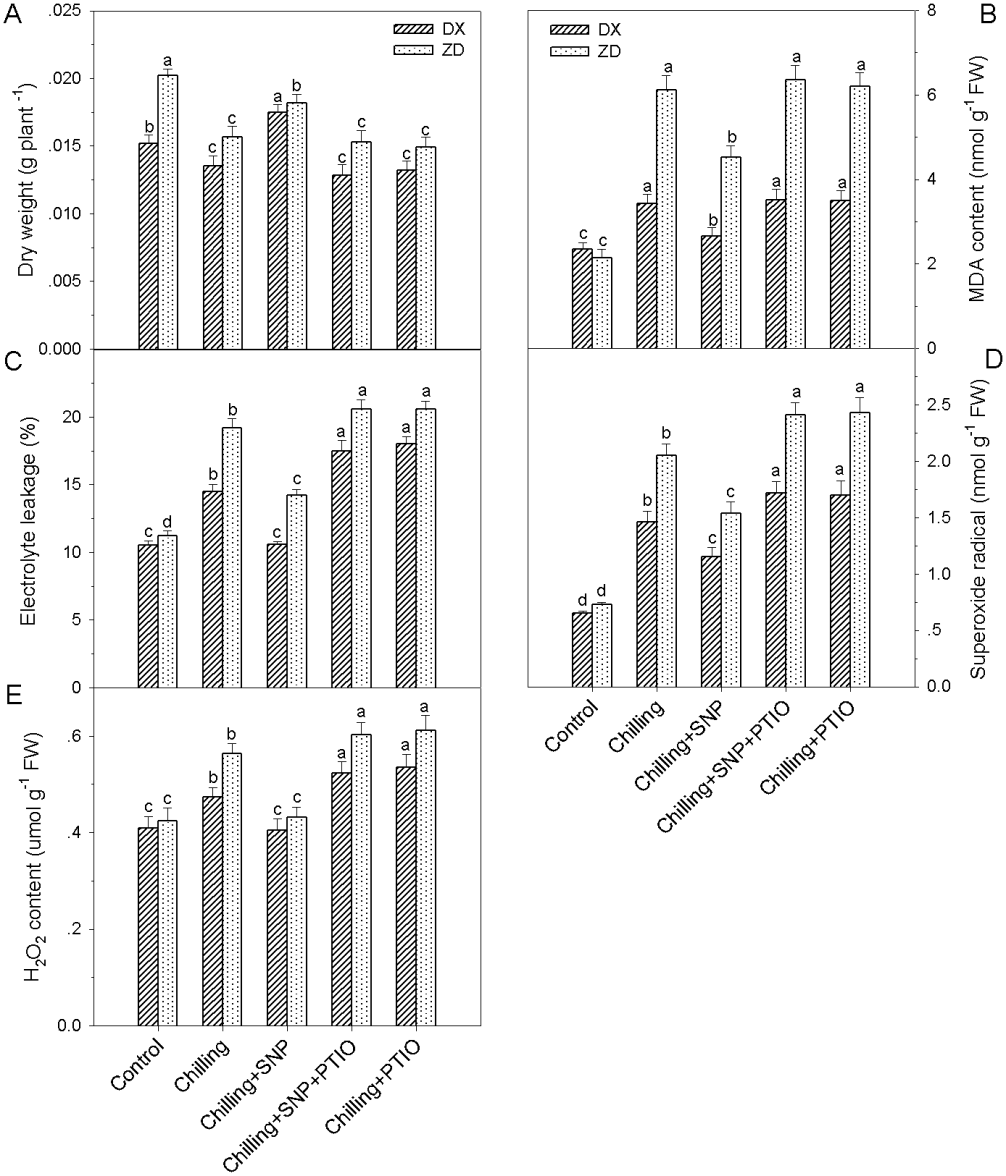
Effects of NO on plant growth and electrolyte leakage, MDA content, H_2_O_2_ and superoxide radical accumulation in leaves of *E*. *nutans* (DX, Damxung and ZD, Zhengdao) under chilling stress. Four-week-old seedlings were pretreated with distilled water, 100 μM SNP, 200 μM PTIO, and 100 μM SNP + 200 μM PTIO for 12 h, respectively, and then exposed to chilling stress (5°C) for 5 d. Plants treated with distilled water under the normal conditions (25°C) served as controls. Each value represents the mean ± SD. Each experiment was repeated at least three times. Bars with different letters are significantly different at the 5% level.

Pretreatment with the NO donor SNP also induced increases (*P* < 0.05) in fresh weight and dry weight of both sources of *E*. *nutans*, and the increases were substantially arrested by the pretreatment with the NO specific scavenger PTIO (Fig 2). On the one hand, PTIO alone had no effect on two varieties of *E*. *nutans* (DX and ZD) under control conditions (data not shown).

### NO mitigates chilling stress-induced oxidative damage

Pretreatment with 100 μM SNP remarkably reduced electrolyte leakage, MDA concentrations and ROS levels in leaves of DX and ZD under chilling stress. Electrolyte leakage, MDA, H_2_O_2_ and superoxide radical decreased (*P* < 0.05) by 27%, 26%, 23% and 2% in ZD compared with under chilling stress alone, respectively, whereas electrolyte leakage, MDA, H_2_O_2_ and superoxide radical restored to control level in DX (Fig 2). The reduction of oxidative damage by SNP treatment was completely blocked by the pretreatment with the NO scavenger PTIO.

### NO is involved in ALA-induced reduction in membrane lipid peroxide levels

**Fig 3:**
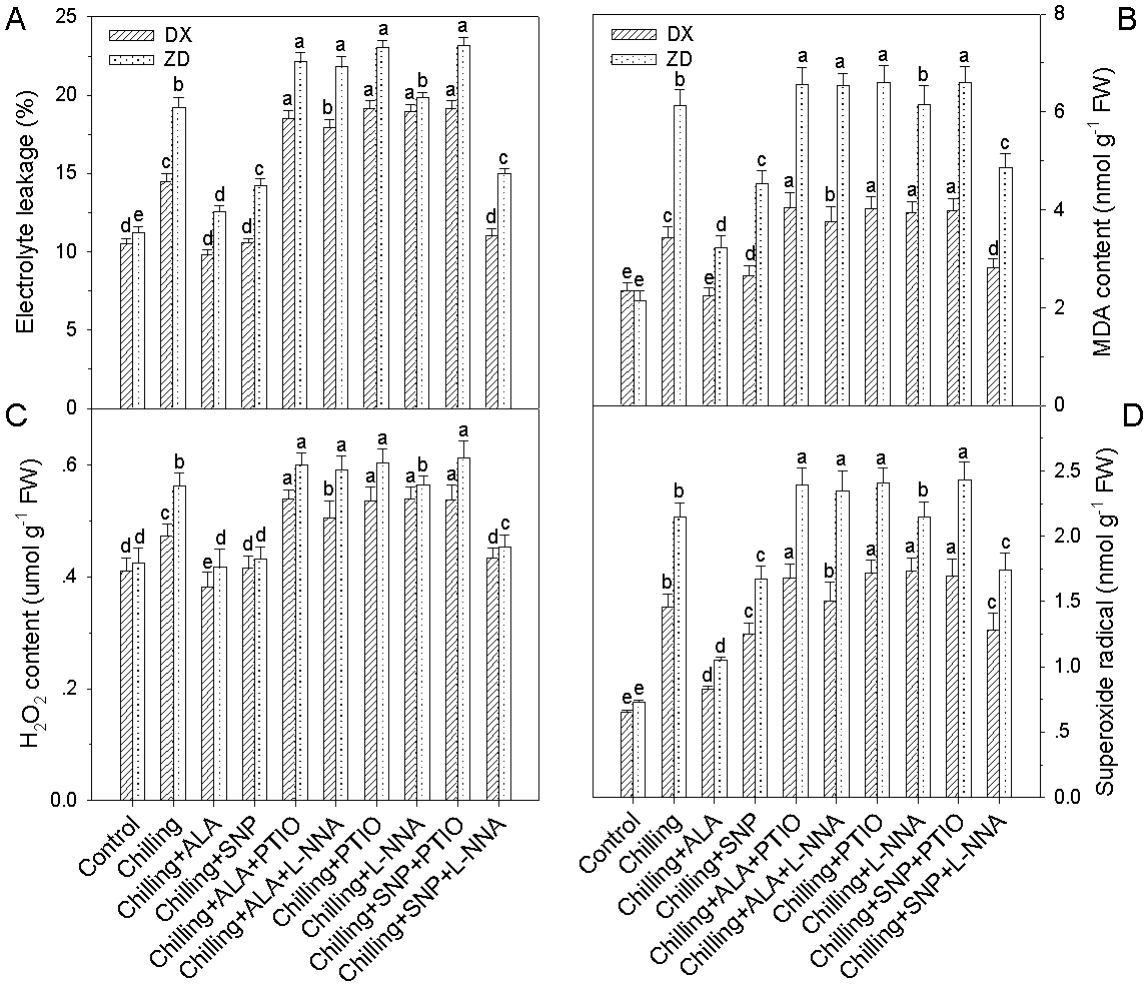
Effect of ALA and NO on electrolyte leakage, MDA content, H_2_O_2_ and superoxide radical accumulation in leaves of *E*. *nutans* (DX, Damxung and ZD, Zhengdao) under chilling stress. Four-week-old seedlings were pretreated with distilled water, 6 μM ALA, 100 μM SNP, 6 μM ALA + 200 μM PTIO, 6 μM ALA + 150 μM L-NNA, 200 μM PTIO, 150 μM L-NNA, 100 μM SNP + 200 μM PTIO, and 100 μM SNP + 150 μM L-NNA for 12 h, respectively, and then exposed to chilling stress (5°C) for 5 d. Plants treated with distilled water under the normal conditions (25°C) served as controls. Each value represents the mean ± SD (n = 3). Bars with different letters are significantly different at the 5% level.

Pretreatments with PTIO and NOS inhibitor L-NNA in the presence of ALA, electrolyte leakage, MDA content and ROS levels rose evidently (Fig 3), but these pretreatments alone did not affect the membrane lipid peroxide levels and concentrations of ROS in the control leaves (data not shown). Moreover, application of L-NNA significantly enhanced electrolyte leakage, MDA content and ROS levels in leaves of DX, but had little effect on leaves of ZD, which indicates that significant activated NOS exists in DX leaves under chilling stress, but not in leaves of ZD, and that application of L-NNA inhibits NOS activity in DX leaves.

### NO is required for ALA-induced antioxidant defense

Pretreatments with PTIO and L-NNA almost blocked (*P* < 0.05) ALA-induced enhancements in the concentrations of GSH, AsA, total glutathione, total ascorbate, and the ratios of reduced/oxidized glutathione (GSH/GSSG) and reduced/oxidized ascorbate (AsA/DHA), and the activities of SOD, CAT, APX and GR, especially in DX leaves, but these pretreatments alone did not affect the concentrations of non-enzymatic antioxidants and the activities of antioxidant enzyme in the control leaves (data not shown). These results suggested that NO is required for ALA-induced the up-regulation of antioxidant defense, resulting in the reduction of ROS accumulation in chilling-stressed *E*. *nutans* leaves.

**Fig 4:**
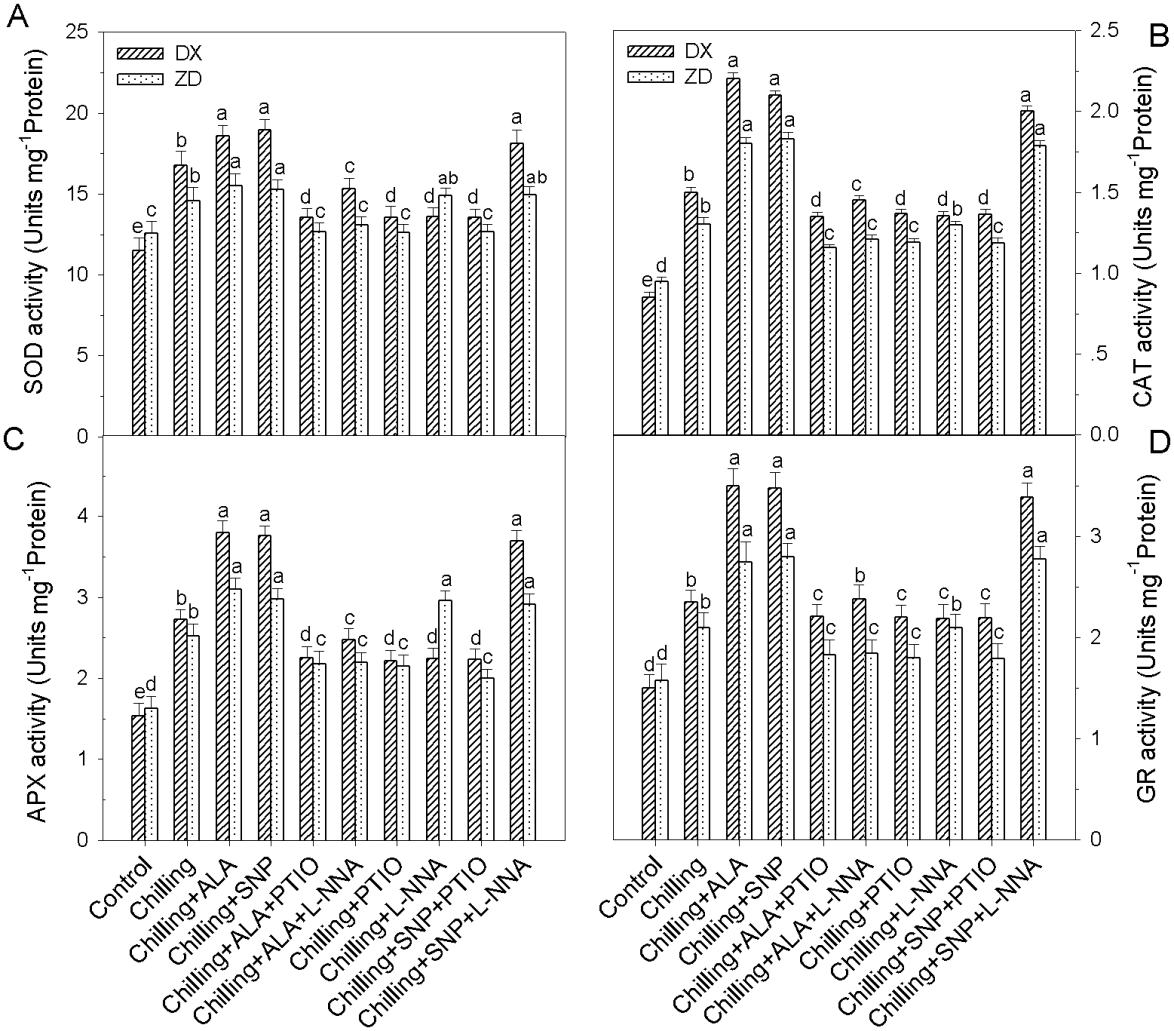
Effect of ALA and NO on the activities of antioxidant enzyme (SOD, CAT, APX and GR) in leaves of *E*. *nutans* (DX, Damxung and ZD, Zhengdao) under chilling stress. Each value represents the mean ± SD (n = 3). Bars with different letters are significantly different at the 5% level.

**Fig 5:**
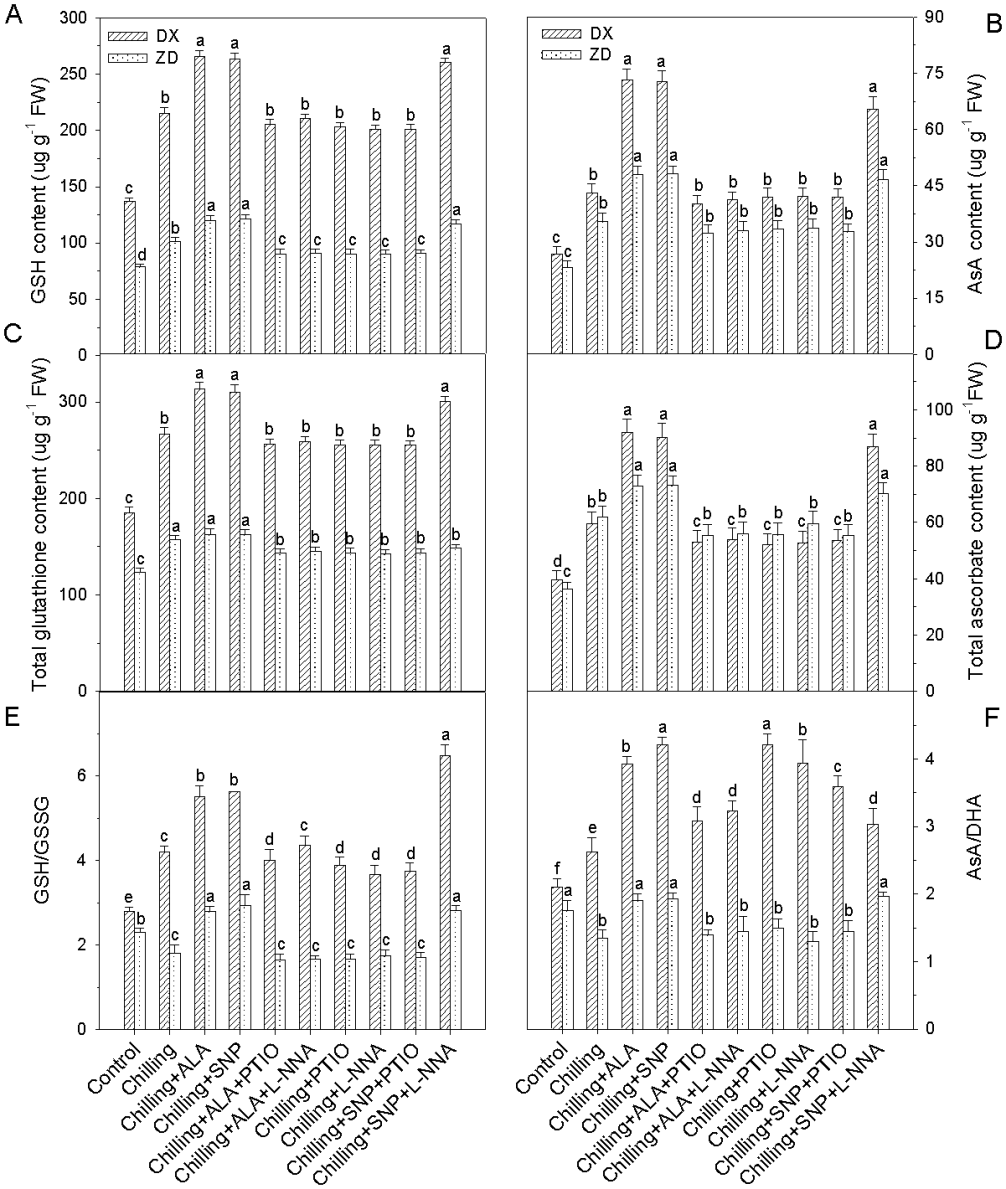
Effect of ALA and NO on contents of non- enzymatic antioxidants (GSH, AsA, Total glutathione and ascorbate) and ratios of GSH/GSSG and AsA/DHA in leaves of *E*. *nutans* (DX, Damxung and ZD, Zhengdao) under chilling stress. Each value represents the mean ± SD (n = 3). Bars with different letters are significantly different at the 5% level.

Moreover, treatment with SNP also induced increases in the non-enzymatic antioxidants and the total activities of antioxidant enzymes, and the increases were substantially inhibited by the pretreatment with PTIO and L-NNA in the leaves of DX (Figs 4 and 5). These results clearly indicate that NO itself can induce the up-regulation of antioxidant defense systems in plants.

### NO mediates ALA-induced activation of Plasma Membrane (PM) H^+^-ATPase

**Fig 6:**
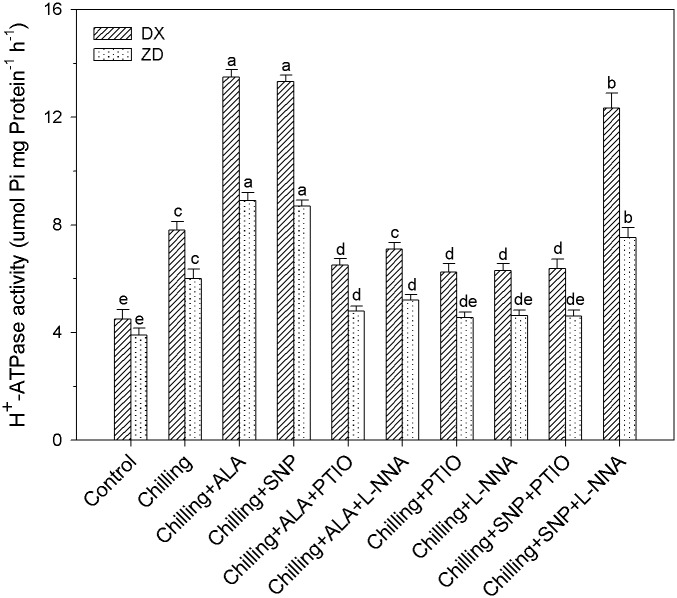
Effect of ALA and NO on activity of H^+^-ATPase in leaves of *E*. *nutans* (DX, Damxung and ZD, Zhengdao) under chilling stress. Each value represents the mean ± SD (n = 3). Bars with different letters are significantly different at the 5% level.

Recent studies suggest that an increase in the activity of PM H^+^-ATPase plays an important role in chilling acclimation [48, 49]. Chilling stress resulted in a 73% and 54% increase (*P* < 0.05) in H^+^-ATPase activity in DX and ZD, compared with control (Fig 6). The activation of PM H^+^-ATPase induced by ALA was inhibited by the pretreatments with PTIO and L-NNA in leaves of *E*. *nutans* plants, suggesting that NO mediates the ALA-induced PM H^+^-ATPase up-regulation. Moreover, SNP treatment led to a rapid activation of the PM H^+^-ATPase. The activation of PM H^+^-ATPase by SNP treatment was completely blocked by the pretreatment with the NO scavenger PTIO. These results indicate that NO itself can induce the activation of PM H^+^-ATPase.

### ALA-induced enhancements in endogenous NO production and NOS activity

**Fig 7:**
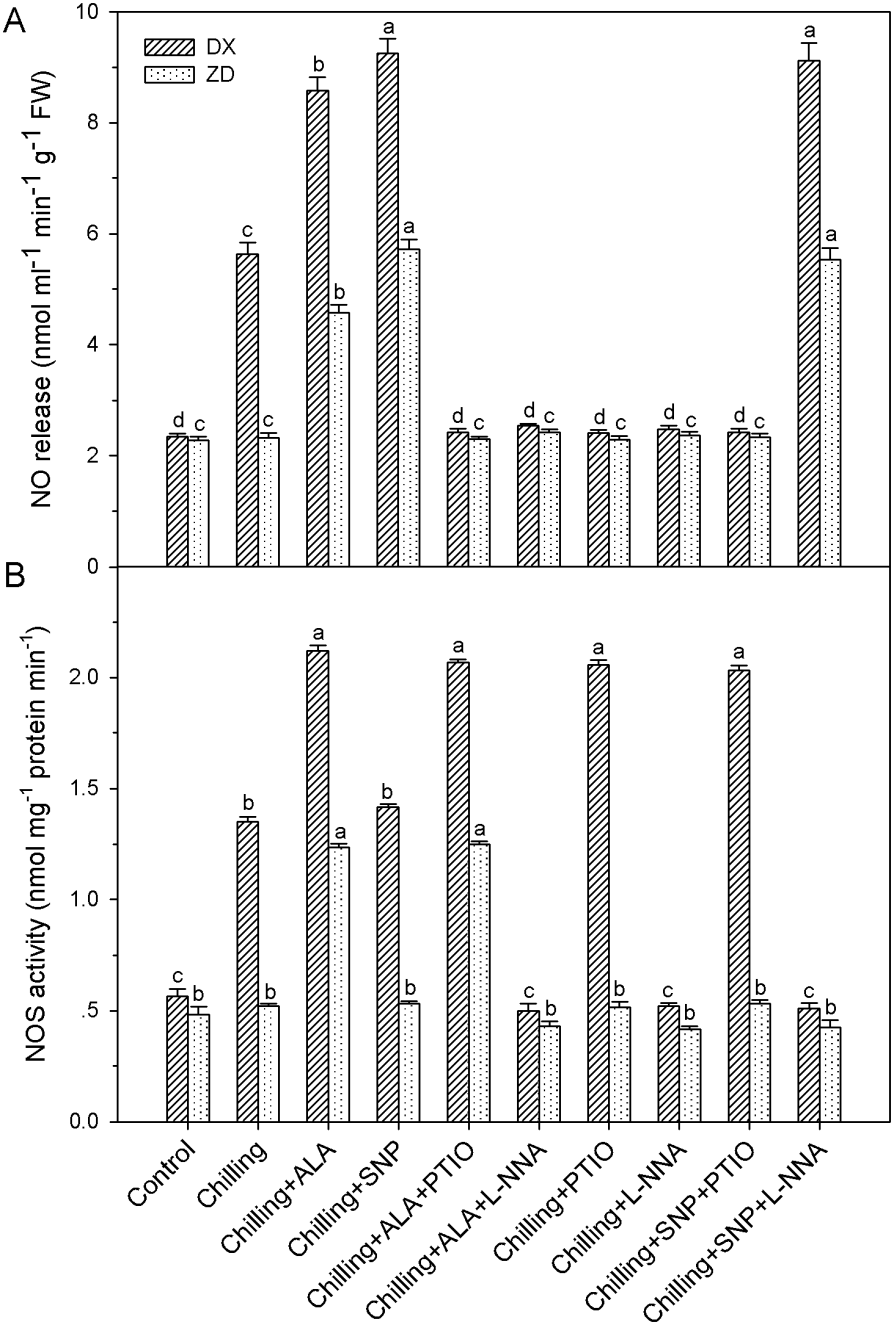
NO production and NOS activity in leaves of *E*. *nutans* (DX, Damxung and ZD, Zhengdao) under chilling stress. Each value represents the mean ± SD (n = 3). Bars with different letters are significantly different at the 5% level.

Treatment with 1 mg l^-1^ ALA led to rapid increases in NO release and activity of NOS, and the increases were blocked (*P* < 0.05) by the pretreatment with PTIO or L-NNA in both plants exposed to chilling stress (Fig 7). These results indicate that exogenous ALA does induce an increased generation of NO in *E*. *nutans*. PTIO and L-NNA was used alone, which had no effect on two varieties of *E*. *nutans* under control condition (data not shown).

To elucidate the possible involvement of nitrate reductase (NR) in ALA-induced NO synthesis, we applied the NR inhibitor Tu (sodium tungstate). Tu did not effect on NO release, either in control or exogenous ALA-treated *E*. *nutans* leaves. NO production remained at the same low level in leaves of both *E*. *nutans* under control, chilling stress alone or ALA treatment in combine with chilling stress (data not shown). These results provide evidence that this NR does not play a role as a source of ALA-induced NO generation.

In contrast, pretreatments with PTIO eliminated (*P* < 0.05) NO accumulation and increases in activity of NOS induced by exogenously applied ALA and chilling stress in the leaves of DX. Comparatively, application of NOS inhibitor L-NNA reduced the activity of NOS as well as NO production in both *E*. *nutans* (Fig 7). These results suggest that NO mediates ALA-induced endogenous NO and activity of NOS in two sources of *E*. *nutans*, and NO synthesis is NOS-dependent in DX expose to chilling stress.

### NO does not induce endogenous ALA synthesis and activation of ALAS

**Fig 8:**
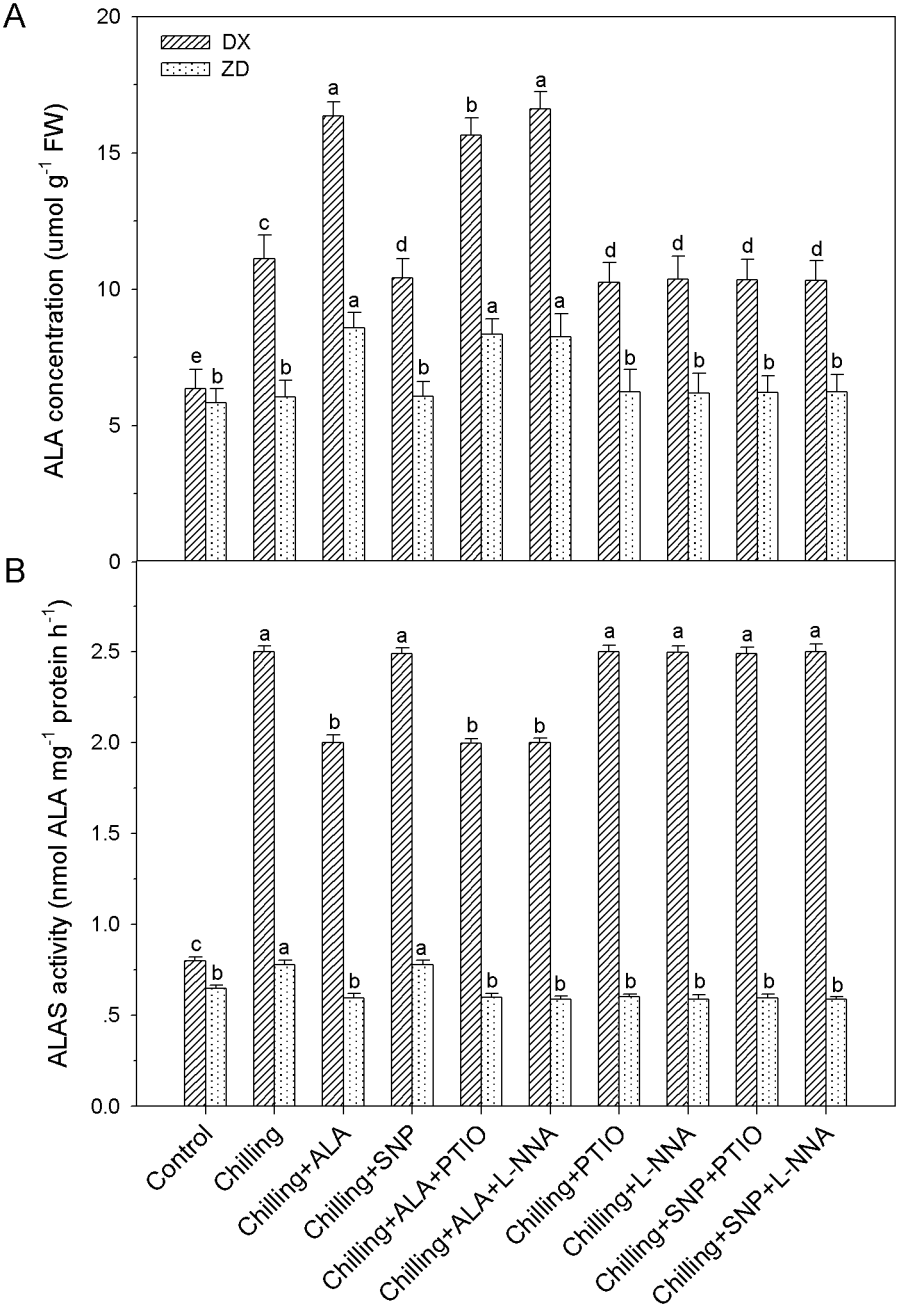
Endogenous ALA content and ALAS activity in leaves of *E*. *nutans* (DX, Damxung and ZD, Zhengdao) under chilling stress. Each value represents the mean ± SD (n = 3). Bars with different letters are significantly different at the 5% level.

Chilling stress caused increases in ALA content, 76% and 3% greater than the control in leaves of DX and ZD, respectively. Pretreatment with exogenous ALA, ALA + PTIO or ALA + L-NNA increased (*P* < 0.05) ALA content in leaves of DX and ZD, respectively. However, application of SNP, PTIO and L-NNA did not affect the endogenous ALA production in the leaves of *E*. *nutans*, indicating that NO does not induce ALA synthesis (Fig 8A). The changed pattern of ALAS was similar to that of endogenous ALA concentration in the leaves of DX and ZD exposed to chilling stress (Fig 8B).

## Discussion

Previous studies have demonstrated that chilling stress-induced oxidative stress led to membrane lipid peroxidation, cellular membranes disruption and growth inhibition [2, 50]. The present study showed that chilling stress resulted in more severe oxidative damage and growth suppression in ZD than in DX, indicating DX were relatively chilling-resistant in comparison with ZD.

ALA, a potential plant growth regulator, has been reported to be involved in many adaptive responses to different biotic and abiotic stresses [12, 15, 16, 18]. Of particular relevance to this work, ALA has increased chlorophyll content, relative water content as well as CAT activity in soybean, which, in turn, enhanced plant resistance to cold stress [13]. In the present study, pretreatment with ALA ameliorated chilling stress by mitigating electrolyte leakage, lipid peroxidation and growth suppression and improved the activities of SOD, CAT, APX, GR and accumulations of AsA and GSH as well as the ratios of GSH/GSSG and AsA/DHA in two sources of *E*. *nutans*. These responses are in agreement with the findings of Korkmaz et al. [14] and Nishihara et al. [51]. In addition, activity of PM H^+^-ATPase in ALA-treated plants was much higher than in plants exposed to chilling treatment alone. Levels of H_2_O_2_ and ${\text{O}}_{2}^{•-}$ were down-regulated, which provided a mechanism by which ALA pretreatment enhanced chilling resistance in *E*. *nutans*. These results suggest that exogenous ALA could elevate the chilling resistance in *E*. *nutans* by enhancing the antioxidant defense system, resulting in reduced ROS accumulation, alleviating oxidative injury caused by chilling stress.

There is ample evidence supporting the regulatory role of NO in plant development and environmental stress response [52–55]. Here, we found that exogenous NO pretreatment could improve chilling resistance in *E*. *nutans*. Acquisition of this chilling resistance involved reduction in ROS accumulation and enhancement of activities of APX, CAT, GR and SOD and the concentrations of AsA and GSH as well as increased activity of PM H^+^-ATPase, which was completely eliminated by PTIO treatment. A similar influence of NO-mediated antioxidant enzyme activities was found in *Baccaurea ramiflora* seeds in which NO treatment reduced H_2_O_2_ accumulation and subsequent damage during chilling stress [56]. Chilling stress enhanced in NO production in the leaves of DX and ZD. Similar results are observed in cold-induced endogenous NO accumulation alleviating cold injury by enhancing antioxidant defense systems in the loquat fruit [57], indicating that an increase in NO concentration is a mechanism protecting *E*. *nutans* from damage induced by chilling stress.

Pharmacological studies have suggested that an NOS-like enzyme and NR are responsible for NO synthesis, although the protein showing NOS-like activity in plants remains unidentified [22, 23, 58, 59]. In this study, Tu did not effect on NO release, either in control or exogenous ALA-treated *E*. *nutans* leaves, which confirmed NR could not catalyze synthesis of NO in the leaves of *E*. *nutans* exposed to chilling stress. Application of L-NNA inhibited the activity of NOS as well as NO generation, which led to oxidative injury. In contrast, Bai et al. [56] detected NR-dependent NO synthesis under chilling stress. The discrepancy regarding the source of NO synthesis between the two studies could be attributed to the difference in the experimental materials used. Our results demonstrated that NOS-derived NO synthesis contributes to enhancement in chilling resistance in *E*. *nutans*, especially in DX.

Although the roles of ALA and NO have been investigated extensively with respect to plant physiological processes under stress conditions, the cross-talk between ALA and NO has remained unexplored. Here, we found that prevention of NO accumulation by PTIO or L-NNA could eliminate the protective effect of exogenous ALA against chilling injury in both plants. These data imply that the protective effect of ALA under chilling stress might be mediated by NO. In addition, application of exogenous ALA increased the NO content and NOS activity in DX under chilling stress, and the increases in the content of NO and the activity of NOS in ALA-treated plants were blocked by the NO scavenger PTIO and NOS inhibitor L-NNA, indicating that ALA could trigger NO accumulation during chilling treatment. Interestingly, neither SNP nor PTIO or L-NNA treatments had an effect on endogenous ALA content in DX leaves. These results illustrate that both ALA and NO are involved in the acquisition of chilling resistance in *E*. *nutans* seedlings, and NO might be a downstream signal in ALA-induced chilling resistance, similar to the previous finding that NO acts downstream of CO to regulate chilling stress [56]. This protective mechanism in chilling-resistant DX was found to more efficient than that in chilling-sensitive ZD.

Earlier reports demonstrated that abscisic acid (ABA), ethylene, calcium ion (Ca^2+^), ROS, salicylic acid (SA) and brassinosteroids (Brs) are also involved in the chilling signal transduction networks [1, 60–63]. Additionally, NO activated mitogen-activated protein kinase (MAPK) and Ca^2+^/CaM-dependent protein kinase (CCaMK) play crucial in antioxidant defense [26, 28]. However, little is known about the interactions between them and the signal pathway under chilling stress. So, further work needs to take a view of signal transduction networks and cross-talk between these components in the ALA signaling transduction pathway in response to chilling stress.

In conclusion, our research indicates that the improvement of exogenous ALA-induced chilling resistance in *E*. *nutans* is associated with an enhancement in the antioxidant defense system and PM H^+^-ATPase activation, depressing the overproduction of ROS to alleviate chilling-induced oxidative damage. NO is involved in ALA-induced antioxidant defense, and that cross-talk between NO and ALA plays a pivotal role in chilling resistance of *E*. *nutans*. Our findings provide further insights into the ALA signaling transduction pathway in plants under chilling stress. However, the complex molecular network operating during chilling treatment mediated by ALA remains to be determined. Further studies are needed to elucidate the cross talk between ALA and other signaling molecules in response to chilling stress.
